# Radiotherapy as a partner for immunotherapy in pancreatic cancer: current landscape and future directions

**DOI:** 10.3389/fonc.2026.1756245

**Published:** 2026-03-19

**Authors:** Zhehan Diao, Yun Wang, Shukun Mu, Xiaofeng Wu, Suchun Yu, Zhongming Wang

**Affiliations:** 1 School of Health Science and Engineering, University of Shanghai for Science and Technology, Shanghai, China; 2 Department of Radiation Oncology, Shidong Hospital, Yangpu District, Shidong Hospital Affiliated to University of Shanghai for Science and Technology, Shanghai, China; 3 Department of Pharmacy, Shidong Hospital, Yangpu District, Shidong Hospital Affiliated to University of Shanghai for Science and Technology, Shanghai, China

**Keywords:** Pancreatic ductal adenocarcinoma, immunotherapy combination strategies, immune checkpoint inhibitors, tumor microenvironment; radiotherapy, cancer vaccines

## Abstract

Pancreatic ductal adenocarcinoma (PDAC) continues to rise in incidence and remains one of the deadliest solid tumors due to its insidious onset and extremely poor overall survival. Current therapeutic options—surgery, chemotherapy, and radiotherapy—offer limited benefit for most patients, largely because of the tumor’s dense stromal architecture, profoundly immunosuppressive microenvironment, and inherent resistance to conventional treatments. In recent years, advances in immune checkpoint inhibitors (ICIs), cancer vaccines, oncolytic viruses, and adoptive cell therapies have driven steady progress in the field of PDAC immunotherapy. At the same time, multimodal strategies that combine immunotherapy with radiotherapy, targeted agents, or chemotherapy have begun to show promising synergistic activity. This review summarizes the recent developments in both single-agent and combination immunotherapeutic approaches for PDAC, and highlights the key challenges and future directions for improving treatment outcomes.

## 1 Introduction

PDAC is a highly aggressive and difficult-to-treat malignancy of the digestive system, often presenting with non-specific early symptoms. It is associated with a five-year overall survival rate of less than 10% (1). Surgical resection remains the only potentially curative treatment; however, only approximately 20% of patients are eligible for radical resection at the time of diagnosis, as most present with locally advanced or metastatic disease (2). Conventional treatment strategies have relied heavily on systemic chemotherapy. Although multi-agent regimens such as FOLFIRINOX or gemcitabine combined with nab-paclitaxel have improved survival outcomes, their overall efficacy remains limited, with a median overall survival (mOS) typically ranging from 9 to 12 months (3, 4). Consequently, developing novel therapeutic strategies is a major priority in both clinical and research settings.

The advent of cancer immunotherapy has fundamentally reshaped the treatment landscape for multiple malignancies. Immune checkpoint inhibitors (ICIs), which target inhibitory pathways such as programmed cell death protein 1 (PD-1), programmed death-ligand 1 (PD-L1), and cytotoxic T-lymphocyte–associated protein 4 (CTLA-4), have demonstrated durable clinical benefits in cancers including melanoma, non-small cell lung cancer, and renal cell carcinoma (5). In contrast, PDAC has shown minimal responsiveness to immunotherapy, with objective response rates to single-agent ICIs generally below 5%. Meaningful clinical responses are largely restricted to a small subset of patients—less than 2%—harboring high microsatellite instability-high (MSI-H) or mismatch repair deficiency (dMMR) (6). The limited efficacy of immunotherapy in PDAC has been attributed to multiple factors, including low tumor mutational burden, defective antigen presentation, insufficient effector T-cell infiltration, and a profoundly immunosuppressive tumor microenvironment (TME) (7). Collectively, these features characterize PDAC as a classic “immune-cold” tumor, characterized by low effector T-cell infiltration and a highly immunosuppressive TME, which poses a major barrier to effective immune-mediated tumor control (8).

In light of these challenges, it is increasingly recognized that single-agent immunotherapeutic approaches are unlikely to achieve substantial clinical benefit in PDAC, thereby underscoring the need for rational combination strategies. A range of multimodal approaches has emerged from early-phase clinical investigations, including therapeutic cancer vaccines (such as GVAX and ELI-002), CD40 agonists (e.g., mitazalimab), and combinations of ICIs with chemotherapy or radiotherapy. In parallel, advances in personalized neoantigen vaccines and mRNA-based platforms have further expanded the therapeutic landscape (9).

Among these strategies, radioimmunotherapy—the combination of radiotherapy and immunotherapy—has garnered particular interest. Radiotherapy can enhance tumor immunogenicity by inducing immunogenic cell death, promoting antigen release and presentation, and activating innate immune signaling pathways such as the stimulator of interferon genes (STING) pathway (10). Moreover, recent technological innovations, including magnetic resonance–guided adaptive radiotherapy (MRgRT), enable the safe delivery of high biologically effective doses (BED), thereby providing a robust technical foundation for the integration of radiotherapy with immunotherapeutic approaches in PDAC (11).

Given the limited success of immunotherapy alone in PDAC, combination strategies—particularly those integrating radiotherapy—have emerged as a promising avenue. In this review, we focus on the rationale and clinical evidence for radio-immunotherapy in PDAC. We summarize the biological mechanisms underlying immune resistance in PDAC, including the critical role of the TME, and discuss how radiotherapy may function as a key modulator to enhance antitumor immune responses. In addition, we review recent clinical advances, emerging combination strategies, and technological developments in radiotherapy delivery, while highlighting the major challenges and future directions for the development of effective radioimmunotherapy approaches in PDAC.

future directions for the development of effective radioimmunotherapy approaches in PDAC.

## 2 Tumor microenvironment

PDAC is characterized by a profoundly immunosuppressive TME and is widely regarded as a prototypical “cold tumor,” marked by limited effector T cell infiltration and resistance to immunotherapy (12, 13). This hostile immune landscape plays a central role in therapeutic resistance and represents a major biological barrier to effective immunomodulatory strategies.

The immune compartment of the PDAC TME is dominated by immunosuppressive cell populations. Tumor-associated macrophages (TAMs), predominantly exhibiting an M2-like phenotype, promote immune evasion through the secretion of anti-inflammatory cytokines such as interleukin-10 (IL-10) and transforming growth factor-β (TGF-β), thereby impairing cytotoxic T cell function. In parallel, myeloid-derived suppressor cells (MDSCs) inhibit T cell activation through metabolic reprogramming and nutrient depletion, while regulatory T-Cells (Tregs) further enforce immune tolerance within the tumor milieu (12).

Beyond immune cells, non-immune stromal components critically shape immune exclusion in PDAC. Cancer-associated fibroblasts (CAFs) constitute a major fraction of the tumor mass and generate a dense desmoplastic stroma that restricts immune cell infiltration. Notably, CAF-derived chemokines such as CXCL12 have been shown to spatially exclude T cells from the tumor core, thereby reinforcing immune privilege and limiting the efficacy of immune checkpoint blockade (14).

At the molecular level, PDAC tumors frequently exhibit elevated expression of immunosuppressive mediators, including TGF-β, IL-10, and indoleamine 2,3-dioxygenase 1 (IDO1), which collectively dampen antitumor immunity and promote T cell dysfunction. These signaling pathways have been directly implicated in resistance to ICIs, particularly in microsatellite-stable (MSS) PDAC (15, 16).

In addition to myeloid and T cell populations, emerging evidence suggests a context-dependent role for B cells in the PDAC TME. Distinct B cell subsets, particularly regulatory B cells (Bregs), have been shown to exert immunosuppressive effects through the secretion of cytokines such as interleukin-10 (IL-10) and transforming growth factor-β (TGF-β), thereby impairing cytotoxic T cell activity and promoting tumor progression (17). Conversely, certain B cell populations may also contribute to antitumor immunity under specific conditions, highlighting the functional heterogeneity of B cells in PDAC. Importantly, these observations have prompted interest in therapeutic strategies aimed at modulating or targeting B cells to alleviate immunosuppression and enhance responses to immunotherapy (17).

Collectively, the immunosuppressive and stromal-rich TME of PDAC provides a strong biological rationale for combination strategies aimed at remodeling immune suppression, enhancing immune cell infiltration, and restoring antitumor immune activity. This framework underpins current efforts to integrate immunotherapy with complementary treatment modalities, including radiotherapy, to achieve synergistic therapeutic effects (18).

## 3 Immunotherapy

### 3.1 ICIs

ICIs are a cornerstone of modern cancer immunotherapy. They function by blocking the PD-1/PD-L1 or CTLA-4 pathways, thereby releasing the suppression of effector T cells in the TME and restoring antitumor immune responses (19). However, in pancreatic ductal adenocarcinoma (PDAC), the clinical benefit of ICIs as monotherapy is largely confined to a small molecularly defined subset of patients with MSI-H/dMMR tumors. While ICIs can effectively activate T cell-mediated antitumor immunity in cancers like melanoma and non-small cell lung cancer (NSCLC), their efficacy as monotherapy in PDAC is markedly limited (6). The KEYNOTE-158 trial reported an objective response rate (ORR) of approximately 18% with pembrolizumab in patients with MSI-H/dMMR PDAC, with a median duration of response exceeding 20 months; however, this subgroup accounts for only 1–2% of the overall PDAC population (20). Importantly, emerging real-world evidence has further confirmed that patients with MSI-H/dMMR PDAC may achieve clinically meaningful and durable responses to ICIs, supporting the translational relevance of these findings beyond clinical trial settings (21, 22). In contrast, in the majority of patients with microsatellite-stable (MSS) PDAC, the efficacy of ICIs is negligible, with monotherapy using durvalumab or pembrolizumab yielding ORR close to 0% and a median progression-free survival of less than three months (23). Therefore, given the constrained efficacy of ICI monotherapy in PDAC, combination strategies have become the primary focus of current research. Among these, combining ICIs with radiotherapy is particularly attractive, as radiation can enhance antigen release and reshape the TME, potentially converting immunologically “cold” tumors into “hot” ones responsive to checkpoint blockade.

### 3.2 Cancer vaccines

Cancer vaccines aim to induce immune recognition and clearance of tumor-associated antigens (TAAs) or tumor-specific antigens (TSAs), and they are considered an important strategy for overcoming the “cold” immune phenotype in PDAC (24). Unlike ICIs, the key advantage of vaccine therapy lies in its ability to actively activate tumor-specific T cells, thereby compensating for the low mutational burden and insufficient antigen presentation in PDAC (24).

Early explorations mainly focused on peptide vaccines and dendritic cell (DC) vaccines. GVAX is a whole-cell vaccine composed of genetically modified human pancreatic cancer cell lines that secrete granulocyte–macrophage colony-stimulating factor (GM-CSF) to enhance antigen presentation (25). A phase I/II clinical trial demonstrated that GVAX could induce antitumor-specific T cell responses and significantly increase tumor-infiltrating lymphocytes (26). In a study combining GVAX with low-dose cyclophosphamide, the median overall survival (OS) of advanced PDAC patients reached 6.7 months, and the disease stabilization rate (SD) was 24% (27). However, subsequent phase III trials (NCT02004262) failed to show an improvement in OS, suggesting that single vaccine therapy still has limitations in clinical translation (28).

Another cell-based vaccine, algenpantucel-L (HyperAcute-Pancreas), is based on allogeneic tumor cells expressing human α-1,3-galactosyltransferase, which induces a strong immune rejection response to promote antitumor immunity (10). Phase II trials showed a 12-month disease-free survival (DFS) rate of 62%, but in subsequent phase III trials, it failed to outperform standard therapy (29). These results indicate that traditional vaccines struggle to exert sustained effects in the immunosuppressive microenvironment of PDAC, providing direction for the exploration of personalized vaccines and combination therapies (30).

In recent years, the development of mRNA technology has made personalized vaccines a key direction in PDAC research. The patient-specific KRAS mutation-based neoantigen vaccine, ELI-002 (which includes KRAS G12D/G12R peptide fragments and AMP adjuvant), demonstrated significant immune activity in the phase I/II AMPLIFY-201 trial (NCT04853017). This study enrolled 25 patients with KRAS mutations in pancreatic and colorectal cancer, and ELI-002 induced a polyclonal KRAS-specific T cell response in over 84% of patients, with detectable tumor antigen-specific amplification in peripheral blood. The 12-month recurrence-free survival (RFS) reached 84%. These results suggest that the personalized KRAS vaccine effectively activates antitumor immunity in the minimal residual disease state, providing potential value for adjuvant therapy. A study using BNT122 (autogene cevumeran) in combination with the anti-PD-L1 monoclonal antibody atezolizumab showed that 50% of the 16 patients generated a strong CD8^+^ T cell response to individualized neoantigens, and patients with a response had significantly extended RFS. Further transcriptomic analysis indicated that the vaccine induced activation of the IFN-γ signaling pathway within the tumor and increased CD8^+^ T cell infiltration, promoting the transformation of the “cold” tumor phenotype into a “hot” one (24).

In the DC vaccine field, personalized vaccines targeting antigens such as WT1, MUC1, and KRAS have gradually shown clinical activity. In a phase II study, a WT1 peptide vaccine combined with chemotherapy extended the median OS of patients to 10.5 months, significantly longer than the 6.5 months seen with chemotherapy alone (31). The OCV-C01 multi-peptide vaccine also showed the ability to induce high levels of WT1-specific T cell expansion and demonstrated good tolerability (32). These results suggest that multi-antigen, personalized vaccine designs may be more suited to PDAC’s immune low-response characteristics.

Overall, cancer vaccines in PDAC are gradually shifting from “immune activation” to “immune remodeling.” Next-generation vaccines, especially KRAS mutation vaccines and personalized mRNA vaccines, not only induce specific T cell responses but also enhance IFN-γ signaling and antigen presentation, driving the conversion of the immune phenotype from “cold” to “hot.”This mechanism lays a solid foundation for combining vaccines with ICIs and other immune modulators, including radiotherapy, which may further enhance antigen presentation and T cell priming.

### 3.3 Adoptive cell therapy

Adoptive cell therapy (ACT) involves the ex vivo expansion and reinfusion of tumor-specific immune cells to enhance the body’s antitumor immune response. It is a key component of current cancer immunotherapy. The main forms of ACT include Chimeric Antigen Receptor T-cell (CAR-T) therapy, T-cell Receptor-engineered T-cell (TCR-T) therapy, Tumor-Infiltrating Lymphocyte (TIL) therapy, and Natural Killer (NK) cell therapy (33).

While CAR-T cell therapy has achieved breakthrough success in hematologic malignancies, its clinical efficacy in PDAC remains significantly limited due to the immunosuppressive TME, tumor stromal barriers, and antigen heterogeneity (34). Research on CAR-T therapy in PDAC has mainly focused on targeting antigens such as mesothelin, Claudin18.2, CEA, and HER2. In an early phase I study, Beatty et al. reported that mesothelin-targeted CAR-T was safe in metastatic PDAC patients, with no dose-limiting toxicities (DLT) observed. However, the ORR was only 7%, and only some patients achieved transient disease stability (35). Subsequent improvement strategies included local infusion or regional arterial infusion to increase the concentration of CAR-T cells within the tumor. In some cases, tumor shrinkage and a decrease in tumor markers were observed (36, 37). Claudin18.2 is another important target, and in a phase I/II clinical trial (NCT04404595, NCT03874897) (37), Claudin18.2-targeted CAR-T treatment achieved a median progression-free survival (PFS) of 4.2 months, with some patients maintaining remission for more than 6 months (32, 38). HER2-targeted CAR-T, however, has higher off-target risks and remains in the early stages of validation (39).

In addition to T cell–based adoptive immunotherapies, chimeric antigen receptor macrophages (CAR-Ms) have recently emerged as a novel and promising cellular platform for the treatment of solid tumors, including pancreatic ductal adenocarcinoma. Unlike CAR-T cells, macrophages are innate immune cells with a natural capacity to infiltrate dense stromal regions and persist within the immunosuppressive TME characteristic of PDAC. Preclinical studies have demonstrated that genetically engineered CAR-Ms can recognize tumor-associated antigens, enhance tumor cell phagocytosis, and actively remodel the TME by promoting a pro-inflammatory macrophage phenotype. Importantly, CAR-Ms have been shown to improve antigen presentation and stimulate endogenous T cell responses, thereby bridging innate and adaptive immunity and potentially overcoming immune exclusion in “cold” tumors. These unique biological properties suggest that CAR-M–based therapies may address key limitations of CAR-T cells in PDAC and represent a complementary strategy for stromal-rich, immune-resistant malignancies. Moreover, CAR-Ms may serve as an attractive component of future combination regimens, including radiotherapy and immune checkpoint blockade, to enhance antitumor immune activation (40).

NK cells, which have innate immune activity independent of antigen presentation, can directly lyse tumor cells and modulate T cell functions in PDAC models (41). A phase I clinical trial (NCT03941457) demonstrated that IL-15 pre-activated NK cell infusion was safe in advanced PDAC, with a median PFS of approximately 3.8 months (42). Furthermore, non-traditional immune cell types, such as γδ T cells, MAIT cells, and CIK cells, have shown some potential in preclinical stages (43).

Although ACT is still in the early exploratory phase for PDAC, its development is increasingly leaning towards engineering and combination therapies. On the one hand, gene-editing technologies (e.g., CRISPR/Cas9) can enhance cell resistance to immunosuppression and improve tumor infiltration (44). On the other hand, combining ACT with ICIs, CD40 agonists, or radiotherapy is believed to significantly extend T cell survival and effector function (45).

### 3.4 Other immunotherapies

In recent years, immune intervention research for PDAC has expanded into areas such as oncolytic virotherapy, cytokine therapy, and novel immune delivery systems (46). These strategies aim to improve the immunosuppressive state of the TME by inducing immunogenic cell death, enhancing antigen presentation, and increasing T cell infiltration, thereby providing a stronger foundation for combination immunotherapy (47).

Oncolytic virotherapy is one of the most prominent approaches, which selectively infects and lyses tumor cells, releasing tumor-associated antigens and activating both innate and adaptive immune responses (48). Currently, T-VEC (talimogene laherparepvec) is the only oncolytic virus approved for clinical use. While its efficacy in PDAC remains limited, it has established a foundation for future combination therapies in terms of safety and delivery models (48). Recent clinical studies have shown that oncolytic adenovirus LOAd703, expressing CD40L and 4-1BBL, when combined with the PD-L1 inhibitor durvalumab and chemotherapy, demonstrates good safety and immune activation potential (48, 49). Furthermore, the combination of pelareorep (oncolytic reovirus) with the PD-1 inhibitor pembrolizumab has shown improvements in ORR and progression-free survival (PFS) in certain patients (50). Another study using endoscopic ultrasound (EUS)-guided T-VEC injection in PDAC patients has validated its feasibility and tolerability, providing a new direction for local immune activation (48, 51). While monotherapy with oncolytic viruses has limited efficacy in PDAC, their combination with ICIs or radiotherapy can demonstrate powerful immune-stimulatory effects.

In addition to oncolytic virotherapy, cytokine therapy is considered a key approach to enhancing the immune microenvironment. Recombinant cytokines such as IL-2, IL-15, and IFN-α can promote the activation of T cells and NK cells, but traditional high-dose regimens often lead to severe toxic reactions. A recent study showed that the IL-15 superagonist N-803, when used in combination with ICIs in various solid tumors, significantly enhanced CD8^+^ T cell responses (48). Another next-generation IL-2 derivative, Nemvaleukin alfa, selectively activates effector T cells while avoiding Treg expansion, demonstrating improved safety and tolerance in several phase I/II trials (52). With advances in delivery technology, new immune molecules and viral vectors are being integrated into nanoparticle, liposome, and hydrogel platforms for localized, efficient release while minimizing systemic toxicity. A recent study indicated that IL-12 nanogels significantly enhanced major histocompatibility complex class I (MHC-I) expression and CD8^+^ T cell infiltration in a pancreatic cancer model, improving the immune “cold” tumor state (53). Taken together, multiple immunotherapeutic strategies have been investigated in PDAC, encompassing immune checkpoint inhibition, cancer vaccines, adoptive cell therapies, and emerging combination approaches (**Table 1**). Radiotherapy, by inducing immunogenic cell death and modulating the TME, may synergize with these approaches and is therefore a key component of many ongoing combination trials.

**Table 1:** Overview of immunotherapeutic approaches in pancreatic ductal adenocarcinoma.

Strategy category	Representative agents/approaches	Mechanism of action	Development status/key notes
Immune checkpoint inhibitors	Anti–PD-1, Anti–PD-L1, Anti–CTLA-4	Release inhibitory immune checkpoints	Limited efficacy in MSS PDAC; benefit mainly in MSI-H/dMMR
Cancer vaccines	GVAX, KRAS neoantigen vaccines, ELI-002	Enhance tumor-specific T cell priming	Early-phase trials; immunogenicity demonstrated
CD40 agonists	Mitazalimab, Selicrelumab	Activate antigen-presenting cells	Promising in combination with chemotherapy
Adoptive cell therapy	CAR-T, TCR-T, TILs	Direct tumor targeting by engineered T cells	Early clinical development; TME barriers remain
Oncolytic viruses	Pelareorep	Induce immunogenic tumor cell death	Limited responses; immune activation observed
Combination strategies	RT + ICIs, chemo-immunotherapy	Synergistic immune modulation	Active area of investigation

PD-1, programmed cell death protein 1; PD-L1, programmed death-ligand 1; CTLA-4, cytotoxic T-lymphocyte–associated protein 4; MSS, microsatellite stable; MSI-H, microsatellite instability–high; dMMR, mismatch repair deficiency; PDAC, pancreatic ductal adenocarcinoma; KRAS, Kirsten rat sarcoma viral oncogene homolog; CAR-T, chimeric antigen receptor T cells; TCR-T, T-cell receptor–engineered T cells; TILs, tumor-infiltrating lymphocytes; TME, tumor microenvironment; RT, radiotherapy; ICIs, immune checkpoint inhibitors.

In addition to the strategies discussed above, several other immunomodulatory approaches have been explored in PDAC, although their initial promise has been tempered by subsequent clinical experience. Electroporation, particularly irreversible electroporation (IRE), has gained attention for its ability to induce immunogenic cell death and modulate the tumor microenvironment, thereby potentially enhancing antitumor immunity (54). However, early enthusiasm has been dampened by mixed clinical results and the realization that its immunomodulatory effects may be context-dependent (55). Similarly, CCR2 inhibition, which targets the recruitment of immunosuppressive macrophages, showed preclinical promise in remodeling the TME and improving T cell infiltration (56). Yet, clinical trials have failed to demonstrate consistent benefit, and the initial hype surrounding this approach has faded (57). These examples underscore the challenges of translating preclinical immune modulation into clinical success in PDAC and highlight the need for careful patient selection and combination strategies—such as with radiotherapy—to unlock meaningful responses.

## 4 Advances in research on combined immunotherapy

### 4.1 ICIs + cancer vaccines

Early vaccine therapy was represented by the GM-CSF overexpressing allogeneic pancreatic cancer cell vaccine GVAX combined with *Listeria monocytogenes* expressing the tumor antigen mesothelin (CRS-207). A phase I/II trial demonstrated that, in the regimen of low-dose cyclophosphamide preconditioning + GVAX + CRS-207, patients who received at least three doses had a median overall survival (OS) of 9.7 months compared to 4.6 months for those receiving only two doses (HR 0.53, P = 0.02) (58). However, in the subsequent randomized phase IIb trial, GVAX + CRS-207 did not significantly outperform chemotherapy (median OS 3.7–5.4 months) (59). These results suggest that while vaccine platforms have the ability to induce immune responses, their standalone effect remains limited unless immune suppression mechanisms are overcome.

In a phase I trial involving 25 high-risk recurrence PDAC or CRC patients, approximately 20 of whom had PDAC, the results showed that ELI-002 induced a KRAS-specific T cell response in 84% of patients, with a median RFS of 16.3 months and a median overall survival (OS) of 28.9 months (follow-up: 26.7 months), significantly longer than historical controls (60) The study also observed that ctDNA conversion was positively correlated with immune response, suggesting that the immune response activated by the vaccine could potentially control the disease.

Personalized mRNA vaccines have also made significant progress in PDAC. In one study, 16 high-risk recurrence PDAC patients were treated with personalized neoantigen vaccines, of which 50% (8 patients) generated strong and durable CD8^+^ T cell responses. In the immune-positive group, the median RFS had not yet been reached, while the immune-negative group had an RFS of 13.4 months, with a significant trend difference (24). This study was the first to demonstrate that mRNA vaccines could induce neoantigen-specific T cell clones in PDAC patients, correlating with delayed recurrence. Additionally, a report on the mRNA-lipoplex vaccine in a small-scale trial (n = 18) observed that CD8^+^ T cell clones persisted for up to 12 months post-injection, with enhanced IFN-γ secretion (61). Although clinical sample sizes are still limited, these studies collectively indicate that precise antigen delivery via the mRNA platform can significantly improve the immune recognition foundation of PDAC, paving the way for combination with ICIs.

### 4.2 ICIs + immunomodulators

PDAC is characterized by its highly immunosuppressive TME, which is rich in CAFs, MDSCs, and Tregs. Together, these components form a “barrier” that resists immune attack. As a result, ICIs alone often fail to effectively activate cytotoxic T cell responses in this environment. Therefore, in recent years, researchers have attempted to reshape the TME using immunomodulators to enhance the efficacy of ICIs.

CD40, a member of the TNF receptor superfamily, can be activated to promote dendritic cell maturation and enhance antigen presentation (48). A phase II randomized trial compared nivolumab + chemotherapy, sotigalimab (APX005M) + chemotherapy, and a combination regimen in first-line metastatic PDAC patients (62). The trial involved 105 evaluable patients, with 1-year overall survival (OS) as the primary endpoint. The results showed that the 1-year OS for nivolumab + chemotherapy was 57.7% (P = 0.006 vs historical control of 35%), while sotigalimab + chemotherapy and the combination group showed 48.1% (P = 0.062) and 41.3% (P = 0.223), respectively. Immunologic multi-omics analyzes of the different combinations indicated that the benefit of the combination was closely related to baseline and treatment-phase tumor/peripheral immune phenotypes, suggesting that CD40 activation could biologically enhance the response to chemotherapy-immune combinations in some patients (62).

The combination of epigenetic modulators and ICIs has also shown important translational and clinical signals. The HDAC inhibitor entinostat can inhibit MDSC-mediated immune suppression and promote dendritic cell activation in mouse models. Based on this mechanism, a phase II study combined entinostat with nivolumab in advanced PDAC (63). This trial (n = 27 evaluable) reported an ORR of 11% (3 partial responses), a median progression-free survival (PFS) of 1.89 months, and a median overall survival (OS) of 2.73 months. A small proportion of patients showed durable responses (with a median duration of 10.2 months), accompanied by immune activation evidence, such as dendritic cell activation and upregulation of inflammatory pathways in the tumor (63). This strategy may induce limited but durable clinical responses, providing a biological foundation for future multimodal combinations with vaccines, CD40 agonists, or metabolic interventions.

New-generation CD40 antibodies (e.g., mitazalimab) have shown high tumor response rates in early studies when combined with intensified chemotherapy (mFOLFIRINOX). If confirmed in larger trials, CD40 agonists may become a feasible clinical pathway (64). Overall, the advantage of CD40 agonists is their ability to enhance antigen presentation and improve CD4/CD8 T cell infiltration in the tumor in the short term. However, their benefit is highly dependent on appropriate dosing schedules and the patient’s baseline immune background. They may also be associated with immune-related toxicities or chemotherapy-enhanced toxicities, necessitating biomarker-based cohort screening and dose optimization (62, 64, 65).

In summary, the introduction of immunomodulators provides a new breakthrough for the efficacy of ICIs in PDAC. CD40 agonists and epigenetic inhibitors have shown promising signs of improving immune activity and partial survival benefits in early trials, while the integration of metabolic-targeted drugs remains in the exploratory phase.

### 4.3 Other immunotherapy combination approaches

In PDAC, the tumor’s low immunogenicity, limited immune cell infiltration, and the highly active tumor stroma and immunosuppressive pathways make single or dual immunotherapy approaches difficult to overcome the “cold tumor” barrier. This challenge has led researchers to explore “multiple combination” strategies involving ≥2 immunotherapeutic approaches or the combination of immunotherapy with traditional therapies (chemotherapy, radiotherapy, biological modulators) to convert the tumor from a “cold” to a “hot” tumor and improve clinical response rates (66).

The core mechanism of the multiple combination strategy is as follows: First, vaccines or oncolytic viruses are used to activate or release tumor antigens, increasing the chances of antigen presentation; second, CD40 agonists, metabolic modulators, epigenetic drugs, and TME interventions are employed to weaken immunosuppressive pathways (e.g., MDSCs, Tregs, metabolic exhaustion) and enhance T cell function; finally, ICIs are used to relieve T cell exhaustion, or combined with radiotherapy/chemotherapy to promote immune cell infiltration and immune activation, forming a “multi-dimensional synergistic antitumor” model (67). For example, if vaccine-induced specific T cells cannot survive or are excluded from the tumor by the stroma, combining with CD40 agonists can improve dendritic cell activity and enhance T cell entry into the tumor; additionally, ICIs can extend the effector time of these T cells.

Recent clinical and early-stage research has demonstrated the feasibility of this approach. For instance, a Phase 1 study combining the CD40 antibody SEA-CD40 with gemcitabine + nab-paclitaxel + pembrolizumab in metastatic PDAC patients showed an ORR of 44% (95% CI 31.5-57.6%) in 27 patients. Another study combining APX005M (sotigalimab) with gemcitabine/nab-paclitaxel ± nivolumab indicated that the regimen was well-tolerated in treatment-naïve metastatic PDAC patients and showed early antitumor activity (67).

In the combination of vaccines and ICIs, small-scale studies have suggested enhanced immune responses. For example, a neoantigen mRNA vaccine induced a T cell response rate of 84% in postoperative PDAC patients, with a trend towards delayed recurrence in a median follow-up of 20 months (68). Ongoing multi-combination platform trials involving “vaccines + ICIs +/or chemotherapy/radiotherapy” are advancing.

Moreover, treatment responses are also influenced by the microenvironment and systemic factors. A study found that patients with a baseline neutrophil-lymphocyte ratio (NLR) >3.1 had a poor prognosis with CD40 combination therapy, suggesting that systemic inflammatory states affect the response to combination treatments (68). Review articles also emphasize that immunotherapy combinations for PDAC should simultaneously address antigen activation, immune modulation, and microenvironment intervention in a three-dimensional manner (69).

While these multi-agent immunotherapy combinations have shown promise, the addition of radiotherapy offers a distinct advantage by locally enhancing antigen release and systemic immune activation, forming the basis for the radio-immunotherapy strategies discussed in the following section.

## 5 Immune mechanisms and clinical data in radioimmunotherapy

### 5.1 Immune mechanisms

**Figure 1:**
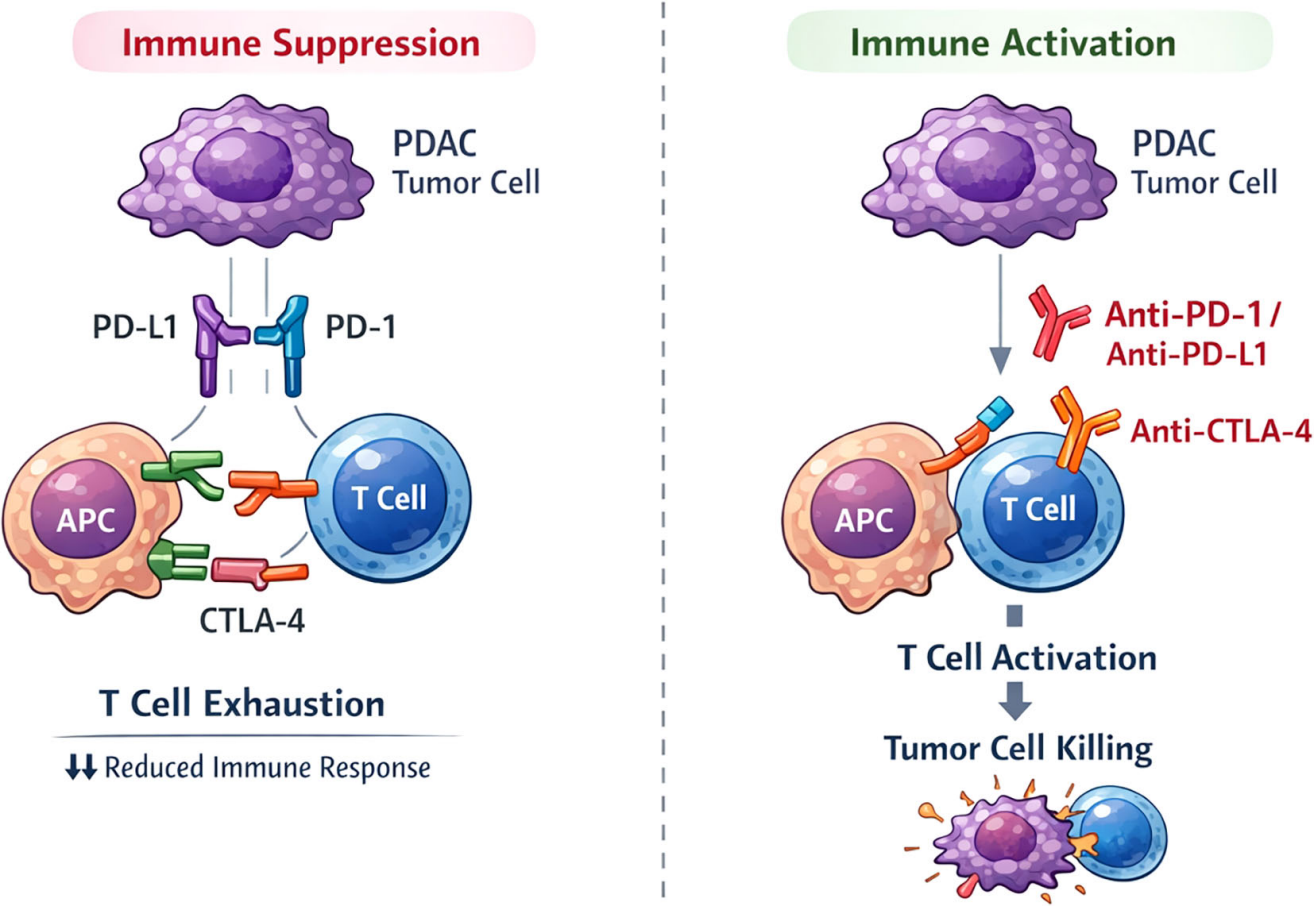
Mechanisms of immune checkpoint inhibition in PDAC. Left panel (Immune Suppression): Tumor cells express PD-L1 (depicted as a transmembrane ligand), which interacts with PD-1 on T cells at the immunological synapse, leading to T cell exhaustion and reduced immune response. CTLA-4 on T cells interacts with CD80/CD86 on antigen-presenting cells (APC), further suppressing T cell activation. Right panel (Immune Activation): Anti-PD-1/PD-L1 and anti-CTLA-4 antibodies block these inhibitory interactions, restoring T cell activation and enabling tumor cell killing. Schematic representation; molecule sizes are exaggerated for clarity and do not reflect actual proportions.

PDAC is one of the most immune-resistant solid tumors, with its TME composed of a dense fibrous stroma, CAFs, MDSCs, and Tregs. This structure physically obstructs immune cells and drugs from entering the tumor core and maintains an immunosuppressive state by secreting factors like TGF-β and IL-10, thereby limiting the function of effector CD8^+^ T cells and NK cells (70, 71). Additionally, PDAC typically has a very low tumor mutational burden (TMB) and limited neoantigen expression, leading to insufficient antigen presentation and reduced immune recognition (72). These features give PDAC a typical “immune cold” phenotype. The immune suppression in PDAC is further exacerbated by the interaction between PD-L1 on tumor cells and PD-1 on T cells, which results in T cell exhaustion and immune evasion. The mechanisms by which ICIs modulate the immune response in PDAC are illustrated in **Figure 1**, which highlights how these inhibitors counteract immune suppression and promote T cell activation.

Radiation therapy (RT), besides directly inducing DNA double-strand breaks, can activate the immune system through immunogenic cell death (ICD). Tumor cells release damage-associated molecular patterns (DAMPs), such as high-mobility group box 1 (HMGB1) and adenosine triphosphate (ATP), after irradiation, and expose calreticulin, which promotes antigen uptake by dendritic cells (DCs) (73). RT can also activate the cGAS-STING pathway, inducing type I interferon (IFN-β) secretion and promoting the activation of CD8^+^ T cells. Animal studies have shown that in pancreatic cancer models, stereotactic body radiotherapy (SBRT) can significantly upregulate MHC-I and PD-L1 expression, increasing CD8^+^ T cell and M1 macrophage infiltration, providing a basis for ICIs (74). Mechanistically, radiotherapy induces immunogenic cell death, leading to the release of damage-associated molecular patterns (DAMPs) such as HMGB1 and ATP, which promote dendritic cell maturation and antigen presentation. Additionally, radiation activates the cGAS-STING pathway in tumor cells and dendritic cells, resulting in type I interferon production and recruitment of CD8+ T cells. These mechanisms collectively contribute to the “*in situ* vaccination” effect of radiotherapy, providing a strong rationale for combining radiation with immunotherapy.

However, the immunosuppressive TME of PDAC can weaken the immune effects induced by radiation. Irradiation can upregulate the expression of PD-L1 and IDO1, thereby triggering new immune evasion pathways (75). RT induces antigen release, “igniting” immune responses, while ICIs lift the immune “brakes,” preventing effector T cell exhaustion (73). Preclinical studies have shown that PD-1 blockade can significantly amplify RT-induced CD8^+^ T cell expansion and generate an abscopal effect (immune response in non-irradiated tumor sites) (76).

RT also enhances immune responses indirectly by modulating tumor metabolism and vascular permeability. Moderate-dose radiation can normalize abnormal blood vessels, improve immune cell infiltration, and reduce lactate accumulation and hypoxia-inducible factor-1α (HIF-1α) levels, thus restoring T cell effector function (77). High-dose SBRT activates the cGAS-STING-IRF3 axis, inducing the secretion of chemokines such as CXCL10 and CCL5, which promote the homing of peripheral T cells. These mechanisms together drive the transition of PDAC from an “immune cold” to an “immune hot” phenotype (78). Despite these immune-stimulatory mechanisms, multiple biological and technical barriers in PDAC can substantially limit the clinical efficacy of radioimmunotherapy, as summarized in **Table 2**.

**Table 2:** Major challenges limiting the efficacy of radioimmunotherapy in pancreatic ductal adenocarcinoma.

Challenge category	Key features	Impact on radioimmunotherapy
Immunosuppressive tumor microenvironment	Abundant CAFs, MDSCs, and regulatory T cells with limited effector T-cell infiltration	Attenuates radiation-induced immune activation and limits the effectiveness of immune checkpoint blockade
Low tumor immunogenicity	Low tumor mutational burden and limited neoantigen expression	Insufficient antigen presentation and weak priming of antitumor T-cell responses
Radiation-induced immune escape	Upregulation of PD-L1 and immunosuppressive enzymes such as IDO1 following irradiation	Counteracts immune-stimulatory effects of radiotherapy and contributes to adaptive immune resistance
Dose delivery and normal tissue constraints	Proximity of the pancreas to radiosensitive gastrointestinal organs	Restricts the safely deliverable radiation dose and limits immune-modulatory potential
Inter-patient heterogeneity	Differences in molecular features and immune status (e.g., MSS vs. MSI-H)	Results in variable treatment responses and challenges treatment standardization

CAFs, cancer-associated fibroblasts; MDSCs, myeloid-derived suppressor cells; PD-L1, programmed death-ligand 1; IDO1, indoleamine 2,3-dioxygenase 1; MSS, microsatellite stable; MSI-H, microsatellite instability–high.

### 5.2 Clinical data

Numerous clinical studies have focused on combining immunotherapy and radiotherapy in PDAC (**Table 3**). These investigations provide important evidence for understanding the potential synergistic effects between immune modulation and radiation, as well as their feasibility in clinical practice. To support the subsequent discussion, we summarize the representative trials that have been completed or are currently underway, thereby outlining the overall research landscape and recent progress in this field.

**Table 3:** Clinical trials evaluating combined immunotherapy and radiotherapy strategies in PDAC.

Trial ID	Phase	Status	Conditions	Enrollment	Treatment approach
NCT02866383	Phase II	Completed	Metastatic/refractory PDAC	~84	SBRT (15 Gy × 1) + Nivolumab ± Ipilimumab
NCT04098432	Phase I/II	Recruiting	Locally advanced/unresectable PDAC post-FOLFIRINOX	15	SBRT followed by Nivolumab
NCT02704156	Phase II	Completed	Locally recurrent PDAC	170	SBRT + Pembrolizumab + Trametinib vs SBRT + Gemcitabine
NCT02648282	Phase II	Completed	Locally advanced PDAC	58	GVAX + low-dose Cyclophosphamide + Pembrolizumab + SBRT
NCT03723915	Phase II	Terminated	Advanced/metastatic PDAC	17	Pelareorep (oncolytic virus) + Pembrolizumab
NCT03104439	Phase II	Recruiting/active	MSS/MSI-H pancreatic cancer	25	Nivolumab + Ipilimumab + Radiotherapy
SMART phase II)	Phase II	Completed	LA/BR PDAC	136	High-BED MR-guided adaptive RT (SMART)

PDAC, pancreatic ductal adenocarcinoma; SBRT, stereotactic body radiotherapy; Gy, gray; MSS, microsatellite stable; MSI-H, microsatellite instability–high; MR, magnetic resonance; BED, biologically effective dose; SMART, stereotactic MR-guided adaptive radiotherapy; LA, locally advanced; BR, borderline resectable.

The NCT02866383 study investigated the efficacy of durvalumab (PD-L1 inhibitor) ± tremelimumab (CTLA-4 inhibitor) combined with SBRT (79). A total of 59 patients with locally advanced PDAC were enrolled, with the primary endpoint being clinical benefit rate (CBR). The results showed that the CBR in the monotherapy group was 17.1%, while in the combination group, it increased to 37.2% (80). Although statistical significance was not achieved, the combination of dual immunotherapy and radiation showed potential in improving tumor control rates in some patients. The incidence of grade ≥ 3 treatment-related adverse events was 24.4% in the monotherapy group and 30.2% in the combination group, indicating overall tolerability.

The NCT04098432 study evaluated the effects of continuing nivolumab maintenance therapy following SBRT (81). The trial enrolled 15 patients with locally unresectable PDAC who had undergone induction FOLFIRINOX treatment. The results showed a median progression-free survival (mPFS) of 8.1 months and a median overall survival (mOS) of 13.0 months, with a 1-year survival rate of 66.7%. The treatment was well tolerated, with no grade 4–5 toxicities observed.

A randomized phase II trial (NCT02704156) compared SBRT + pembrolizumab + trametinib with SBRT + gemcitabine in patients with postoperative locally recurrent PDAC (82). A total of 170 patients were enrolled, and the results showed that the combination therapy group had a median overall survival (mOS) of 14.9 months, compared to 12.8 months in the control group, indicating good survival benefits. The incidence of grade ≥ 3 adverse events in the combination group was 22%, while in the control group, it was 14%, with the main toxicities being hepatic dysfunction and hematologic toxicity. The authors suggested that the combination showed potential to improve survival in KRAS-mutant and PD-L1-positive patients, although toxicity was slightly higher but within tolerable limits.

The NCT02648282 study evaluated the combination of GVAX vaccine, low-dose cyclophosphamide, PD-1 inhibitor pembrolizumab, and SBRT in high-risk or borderline resectable PDAC as neoadjuvant/adjuvant therapy (83). A total of 31 patients were enrolled, and some cases achieved pathological complete response (pCR) post-surgery, accompanied by significant increases in CD8^+^ T cell infiltration. The overall treatment was well tolerated, and no severe perioperative complications were observed (84).

An I/II phase study (NCT03723915) assessed the safety and efficacy of the oncolytic virus Pelareorep combined with the PD-1 inhibitor pembrolizumab in treating recurrent/refractory PDAC. A total of 13 patients were enrolled, and the ORR was 8%, with a CBR of 42%. Immunological analysis showed that the treatment induced the expansion of novel T cell clones and immune activation signals in the TME. There were rare grade ≥ 3 adverse events, and no treatment-related deaths were reported. The researchers concluded that oncolytic viruses could enhance the immunogenicity of PDAC, enabling ICIs to show partial clinical effects even in low-immunoreactive patients (85).

The NCT03104439 study evaluated ipilimumab ± nivolumab combined with SBRT in multiple microsatellite stable (MSS) solid tumors, including a PDAC subgroup (about 22 cases) (86). The results showed that in the PDAC cohort, the ORR was approximately 9%, the disease control rate (DCR) was 36%, and the median PFS was 3.6 months. The incidence of immune-related adverse events (irAEs) was 24%, which was within an acceptable range. The study demonstrated that SBRT enhanced local antigen release and promoted immune effector cell infiltration, but a pure ICI + radiation regimen was insufficient to achieve significant population-level benefits in PDAC.

Magnetic resonance-guided adaptive radiotherapy (MRgRT/SMART) represents a significant advancement in radiation therapy techniques. A phase II study enrolled 136 patients with locally advanced or borderline resectable PDAC and used high biologically effective dose (BED) SMART therapy (87). At a median follow-up of 8.8 months, the 1-year overall survival rate was 65%, and local control was significantly better than historical controls. The incidence of acute ≥3 gastrointestinal toxicity was less than 5%, and no treatment-related deaths occurred. Immune activation signals (peripheral T cell proliferation, increased PD-L1 expression) were observed in some patients. The authors noted that SMART achieved higher dose delivery while ensuring safety, providing a foundation for future combination with ICIs in clinical practice (87).

While SMART represents an important technological advancement in radiation delivery, its role in radioimmunotherapy for PDAC remains investigational. A multi-institutional phase II study of ablative MR-guided adaptive radiotherapy in locally advanced PDAC demonstrated encouraging local control and acceptable toxicity (87). However, most available SMART data derive from single-arm or early-phase studies (88), limiting definitive conclusions regarding survival superiority over conventional SBRT or systemic therapy. Moreover, randomized trials evaluating radiation intensification strategies in PDAC, such as Alliance A021501 and the CROSSFIRE trial (89), have yielded mixed survival results, underscoring the need for cautious interpretation of non-randomized data.

Importantly, the SMART studies were not specifically designed to test combinations with immune checkpoint inhibitors, and thus their immunomodulatory potential in radioimmunotherapy remains inferred rather than directly demonstrated. The optimal radiation dose and fractionation scheme for immune modulation in PDAC also remain undefined (90). In addition, SMART requires specialized infrastructure and expertise, which may limit widespread implementation. Ongoing prospective trials are therefore essential to clarify its true clinical value within radioimmunotherapy strategies.

However, interpretation of the above clinical findings requires careful consideration of methodological heterogeneity across studies. Many of the available data derive from early-phase (Phase I/II) trials primarily designed to assess safety and feasibility rather than powered to detect survival benefit (91). Therefore, limited improvements in overall survival should not be interpreted as definitive evidence of therapeutic failure.

In addition, substantial differences exist in patient populations and study settings. Some trials focus on locally advanced primary tumors, whereas others enroll patients with metastatic disease, often heavily pretreated. Given the distinct biological and immunologic features of primary tumors versus metastatic sites—particularly in the liver—treatment responses may not be directly comparable across studies (92, 93).

Endpoints also vary widely, ranging from objective response rate (ORR) and progression-free survival (PFS) to local control and overall survival (OS). In PDAC, improvements in local control do not necessarily translate into systemic survival benefit, especially in small, non-randomized studies (94). These factors collectively underscore the need for cautious interpretation of existing data and highlight the importance of well-designed randomized trials to clarify the clinical value of radioimmunotherapy (95).

## 6 Discussion

PDAC remains one of the immune therapy-resistant solid tumors. Despite significant breakthroughs in ICIs, tumor vaccines, and radiation combination therapies in other cancers, their efficacy in PDAC remains extremely limited. The root cause of this gap lies in its unique TME and complex immune escape mechanisms. For instance, KEYNOTE-158 showed that pembrolizumab has clear efficacy against MSI-H/dMMR non-colorectal solid tumors, but MSI-H/dMMR is found in only a small proportion of PDAC cases (approximately 1%–2%), and the ORR for single-agent ICIs in most MSS PDAC patients is nearly zero (96).

The immune microenvironment of PDAC is characterized by high-density CAFs and abundant MDSCs, which form physical barriers and secrete immunosuppressive factors such as TGF-β, thereby inhibiting T cell infiltration and effector function. In addition, most PDACs are microsatellite stable (MSS) and low TMB tumors with scarce mutant antigens, making it difficult for the immune system to recognize them.

While chemotherapy and radiotherapy can induce the release of tumor-associated antigens, excessive doses may harm immune cells. Finding a balance between “immune activation” and “immune exhaustion” remains a clinical challenge. Moreover, immune-related adverse events (irAEs) are more complex and unpredictable in multi-drug combination therapies, limiting their clinical applicability. A recent clinical study further enhances the potential pathways to improve PDAC’s response to ICIs. In this biological context, PDAC’s immune resistance involves both primary mechanisms (such as antigen presentation defects and MHC-I downregulation) and acquired mechanisms (such as dynamic upregulation of PD-L1 and metabolic reprogramming leading to T cell dysfunction). Metabolic-related mechanisms (such as lactate accumulation and amino acid pathway alterations) play a crucial role in further inhibiting effective immune responses (97, 98). Additionally, tumor heterogeneity and molecular subtype differences (e.g., stromal-enriched vs. classical types) significantly affect immune infiltration and treatment efficacy, suggesting that precise patient stratification is crucial to enhance trial signal-to-noise ratios (99).

An additional layer of complexity in PDAC immunotherapy lies in the biological heterogeneity between primary tumors and metastatic lesions. The tumor microenvironment of metastases may differ substantially from that of primary tumors in terms of immune cell composition, stromal architecture, and metabolic profile (100). For instance, liver metastases—the most common site of PDAC dissemination—are exposed to a unique hepatic immune milieu characterized by high baseline tolerance, abundant Kupffer cells, and regulatory T cell enrichment, which may promote resistance to immunotherapy (101). Whether the immune and stromal features of primary tumors are recapitulated in metastases has critical implications for treatment resistance and for the design of clinical trials. Most trials enroll mixed populations of patients with localized and metastatic disease, potentially obscuring site-specific treatment effects. Moreover, the efficacy of radioimmunotherapy combinations may differ depending on whether the irradiated lesion is a primary tumor or a metastasis, and whether non-irradiated metastases exhibit the same immune profile to enable abscopal responses (102). Future studies should incorporate multi-site sampling and consider metastasis-specific TME characteristics when evaluating radioimmunotherapy strategies in PDAC.

Recent PDAC research has focused on microenvironment reprogramming, precision stratification, and multimodal combinations. Tumor vaccines (including KRAS-targeted and personalized neoantigen vaccines) have shown promising biological signals in inducing specific T cell responses (103). Targeting myeloid or metabolic pathways (such as CCR2/CCR5 or IDO pathways) or activating antigen presentation chains (such as CD40 agonists) can significantly reprogram the TME. OPTIMIZE-1 (mitazalimab + mFOLFIRINOX) provided promising early clinical evidence, reporting considerable response rates and extended survival signals, suggesting that antigen presentation enhancers combined with standard chemotherapy could be a clinically viable path (104).

Radiation therapy not only induces immunogenic cell death and releases tumor antigens but also activates the cGAS–STING pathway and upregulates chemokines (such as CXCL10), promoting T cell homing. Therefore, radiation is considered an effective tool for “igniting” immune responses. However, radiation can also upregulate inhibitory pathways such as PD-L1 and IDO, explaining the immune escape phenomenon often observed after single-agent radiation therapy. As a result, rational sequencing or combination of radiation therapy with ICIs has become a research focus (105–107).

Technological advancements have provided feasible solutions for optimizing the safety and dosimetry of combination strategies. For example, MR-guided adaptive radiation therapy (MRgRT/SMART) enables higher biologically effective doses (BED) to be delivered while precisely tracking tumor movement, without significantly increasing ≥3 grade toxicities. Short-term immunological monitoring has shown an upregulation of CD8^+^ T cell infiltration and immune activation signals, providing both dosimetric and immunological evidence for integrating with ICIs (87). These findings support the use of higher BED and image-guided adaptive radiation therapy strategies in future trials to amplify immunogenicity while maintaining controllable toxicity.

Beyond the therapeutic strategies discussed above, emerging computational approaches such as artificial intelligence (AI) are being explored to optimize radioimmunotherapy in PDAC. In radiotherapy planning, AI-based algorithms have demonstrated potential to improve dose distribution and reduce toxicity to organs at risk, with some clinical implementation already underway (108). In parallel, AI-driven radiomics—the extraction of quantitative features from medical imaging—is under investigation as a tool to non-invasively characterize tumor heterogeneity and immune phenotypes, which could ultimately aid in patient selection for combination therapies (108).

However, several limitations temper current enthusiasm. First, most AI applications in PDAC radioimmunotherapy remain in early translational stages, with few prospectively validated models. The performance of AI algorithms is highly dependent on training data quality and may not generalize across institutions or imaging protocols (109). Second, the “black box” nature of deep learning models limits clinical interpretability and regulatory acceptance (110). Third, robust integration of multimodal data—imaging, genomic, and clinical—into predictive algorithms remains methodologically challenging (111). Moreover, whether AI-driven personalization translates into improved clinical outcomes has yet to be demonstrated in prospective trials (109).

Thus, while AI offers a compelling framework for enhancing precision in radioimmunotherapy, its clinical utility in PDAC remains to be established. Future studies should focus on developing interpretable, generalizable models and embedding them into prospective clinical decision-support systems to determine whether they can meaningfully improve patient outcomes (112).
